# Correlation between FFP transfusion and standardized mortality ratio in ICU patients

**DOI:** 10.1186/2197-425X-3-S1-A536

**Published:** 2015-10-01

**Authors:** A Vakalos, I Nikitidis

**Affiliations:** Xanthi General Hospital, ICU, Xanthi, Greece

## Introduction

While plasma donation is still necessary as a unique source of human proteins and to treat coagulation disorders, FFP administration seems to have high rate of inappropriate indication. After all, FFP transfusion is not risk free, and is associated with lung injury, infectious disease and circulatory overload in recipients. On the other hand, team of patients who recorded lower standardized mortality ratio assuming better ICU hospitalization efficiency, may record different demand for FFP transfusion than others.

## Objectives

The aim of our retrospective observation study was to test the hypothesis that a correlation exists between FFP transfusion and standardized mortality ratio (SMR) in our both medical and surgical ICU served in community hospital.

## Methods

From January 2006 to June 2014 admitted to our ICU 620 patients, mean age 64.8 years, mean length of ICU stay (LOS) 14.2 days, mean mechanical ventilation duration per ventilated patient (V. Days) 12.23 days, mean APACHE II score on admission 21.2, predicted mortality 38.9%, actual mortality 31.45%, Standardized Mortality Ratio (SMR) 0.80. From our database we looked for SMR and the following values and indexes according FFP transfusion per year from 2006 to 2014 (mean values). Total, per patient, per hospitalization days (HD), per patient under mechanical ventilation (pts V) and per ventilation days (VD) Using linear correlation method, we looked for linear slope, correlation coefficient (r), and coefficient of determination (r^2^), and by linear regression method using ANOVA test we looked for p value, according SMR and FFP transfusion.

## Results

**Table 1:** *Correlation between SMR and FFP.*

FFP	Slope	r	r2	S. Error	Lower C.I.	Upper C.I.	p value
Total	56.763	0.3501	0.1226	57.400	-78.98	192.51	0.3557
Per patient	-0.684	-0.249	0.0621	1.004	-3.060	1.691	0.3176
Per Hosp. Day	-0.018	-0.087	0.0076	0.079	-0.206	0.169	0.8230
Per pt Ventilated	0.5751	-0.199	0.0395	1.701	-3.107	1.957	0.6078
Per Vent Day	-0.058	-0.205	0.0423	0.1049	-0.306	0.1596	0.5952

## Conclusions

According to our data, there was no statistically significant correlation detected between SMR and FFP transfusion indexes. Our data suggest that FFP transfused do not correlate statistically significant with SMR in ICU patients, whatever the ICU hospitalization efficiency achieved.

